# Impact of HIF prolyl hydroxylase inhibitors in heart failure patients with renal anemia

**DOI:** 10.1186/s13104-024-06726-7

**Published:** 2024-03-01

**Authors:** Takahiro Kambara, Rei Shibata, Yuusuke Sakamoto, Teruhiro Sakaguchi, Hiroyuki Osanai, Yoshihito Nakashima, Hiroshi Asano, Toyoaki Murohara, Masayoshi Ajioka

**Affiliations:** 1 Department of Cardiovascular Medicine, Tosei General Hospital, Seto, Japan; 2 Department of Advanced Cardiovascular Therapeutics, Nagoya University Graduate School of Medicine, 65 Tsurumai, Showa, Nagoya, 466-8550 Japan; 3 Department of Cardiology, Nagoya University Graduate School of Medicine, Nagoya, Japan

**Keywords:** Heart failure, HIF-PH inhibitor, NT-proBNP, Renal anemia, Ferritin, TSAT

## Abstract

**Objective:**

Hypoxia-inducible factor prolyl hydroxylase (HIF-PH) inhibitors are a new class of anti-anemia agents. We retrospectively evaluated the safety and efficacy of HIF-PH inhibitors in patients with heart failure (HF) complicated by anemia associated with chronic kidney disase. HIF-PH inhibitor treatment was initiated in 32 patients with chronic HF complicated by renal anemia and were followed up for 3 months.

**Results:**

Hematocrit and hemoglobin levels markedly improved 3 months after HIF-PH inhibitor treatment. However, levels of NT-proBNP, which is an indicator of HF, did not decrease considerably. Based on the rate of change in NT-proBNP, we divided the patients into “responder” and “non-responder” groups. The results showed that considerably more patients had a ferritin level of less than 100 ng/mL in the non-responder group at baseline. There were substantially more patients with TSAT of less than 20% in the non-responder group at 1 month after HIF-PH inhibitor treatment. The cut-off values to maximize the predictive power of ferritin level at baseline and TSAT value at 1 month after treatment were 41.8 ng/ml and 20.75. HIF-PH inhibitor treatment can be expected to be effective for improving both anemia and HF if ferritin≥41.8 ng/ml at baseline or TSAT≥20.75 at 1 month after treatment.

**Supplementary Information:**

The online version contains supplementary material available at 10.1186/s13104-024-06726-7.

## Introduction

Anemia causes organ ischemia to impair cardiac and renal functions. In fact, approximately 60% of patients with heart failure have comorbid anemia, and the prognosis of heart failure complicated by anemia is known to be poor [1]. CKD affects anemia as the kidney produces erythropoietin (EPO), which is a signaling molecule that stimulates erythropoiesis. Patients with anemia increase as CKD progresses and those with severe comorbid anemia are known to have a poor prognosis. Approximately 50% of patients with heart failure have comorbid CKD, and the prognosis of heart failure complicated by CKD is poor [2]. Silverberg et al. proposed cardiorenal anemia syndrome, which is defined as a vicious cycle consisting of such interaction between heart failure and CKD, as well as the pathology of anemia [3]. In patients with heart failure, the incidence of cardiorenal anemia syndrome is 25%. Moreover, cardiorenal anemia syndrome is associated with a higher mortality rate [4]. For prevention of the onset and progression of heart failure, it is extremely important to break the vicious cycle of cardiorenal anemia syndrome at some point. One of methods to achieve this appears to be the treatment of anemia.

In the "RED-HF trial,” which investigated the effects of an erythropoiesis-stimulating agent (ESA) in patients with heart failure complicated by anemia, ESA treatment was not effective for preventing events including cardiovascular death. Instead, the use of ESAs significantly increased the incidence of thromboembolism[5]. In contrast, in the CONFIRM-HF trial, which examined the effects of intravenous iron administration in patients with heart failure complicated by anemia, the hospitalization rate improved considerably [6]. However, to date there have been no reports of favorable effects of oral iron administration to patients with heart failure. Based on these results, the 2021 European Society of Cardiology guidelines recommend screening of patients with chronic heart failure for anemia and iron deficiency [7]. The guidelines also clearly state that intravenous iron administration should be considered to reduce the risk of hospitalization for heart failure in patients with a ferritin level of less than 100 ng/mL or patients with a ferritin level of 100–300 ng/mL and transferrin saturation (TSAT) of less than 20%. As described above, it is imperative to ascertain the severity of anemia and iron deficiency for planning treatment of heart failure.

Hypoxia-inducible factor prolyl hydroxylase (HIF-PH) inhibitors are a new class of anti-anemia agents that inhibit PH activity to stabilize HIF-α and promote the production of endogenous EPO, thereby enhancing the production of hemoglobin (Hb) and erythrocytes [8]. The randomized control study shows that HIF-PH inhibitor is noninferior to ESAs with respect to cardiovascular safety and correction and maintenance of Hb concentrations [9, 10]. However, there is no assessment of HIF-PH inhibitor safety and efficacy for heart failure patients. HIF-α is known to promote the production of endogenous EPO while also having various pleiotropic effects [11]. Particularly in the cardiovascular system, HIF-α is involved in vasomotor regulation through the production of nitric oxide and atrial natriuretic peptide. It promotes angiogenesis mediated by increased expression of vascular endothelial growth factor, and has other effects. Thus, HIF-α is expected to be effective for cardiovascular protection. In this study, we retrospectively evaluated the safety and efficacy of HIF-PH inhibitors in patients with heart failure complicated by renal anemia.

## Main text

### Methods

An expanded Method section can be found in Additional file 3: Methods.

### Study population

We assessed 32 patients with anemia associated with CKD who suffered from heart failure. All participants had a history of treatment for symptomatic heart failure. They were administered oral HIF-PH inhibitors attending the Department of Cardiology in Tosei General Hospital from 2020 to 2021. All patients were Japanese. We excluded super-elderly individuals aged > 90 years, patients undergoing hemodialysis, and those with malignancies.

Anemia associated with CKD and iron deficiency was defined by the criteria of the JSDT Guideline for Renal Anemia in CKD [12]. Patients who had already met these criteria and used ESAs before the start of HIF-PH inhibitor treatment were also determined to have anemia associated with CKD. ESAs were switched to HIF-PH inhibitors only in patients with Hb level < 11 mg/dL and those with Hb level ≥ 11 mg/dL who requested switching from injections to oral medications. Patients who had been taking oral iron preparations before the start of HIF-PH inhibitor treatment continued taking them without any modification. Only during the 3-month follow-up period in this study, treatment was continued without any changes in the doses of iron preparations even if iron dynamics fluctuated. For HIF-PH inhibitors, iron preparations, ESAs, and drugs for heart failure, we did not change the types or doses of the drugs during the 3-month follow-up period.

## Results

### Patient characteristics

A total of 32 elderly patients with renal anemia who were administered oral HIF-PH inhibitors were enrolled in this study. Their baseline characteristics are shown in Table 1. Patients were 82.0 ± 6.3 years of age and 59.0% were male. All study subjects were patients with a history of treatment for symptomatic heart failure. A total of 76.9% of patients had hypertension, 79.4% had dyslipidemia, 48.7% had diabetes. Moreover, 43.6% of patients had coronary artery disease, 38.5% had a history of atrial fibrillations, and 53.8% had a history of stroke. All participants were non-dialysis-dependent patients with CKD.

**Table 1:** Baseline characteristics (n=32)

Age (years)	82.0 ± 6.3
Male/Female	20/12
BMI (kg/cm2)	22.0 ± 3.3
Systolic Diastolic BP (mmHg)	122.6 ± 20.2
Diastolic BP (mmHg)	59.5 ± 11.2
EF (%)	
HFrEF -no.(%)	5 (16)
HFmrEF -no.(%)	4 (13)
HFpEF -no.(%)	23 (72)
Laboratory data	
Albumin (g/dl)	3.7 ± 0.5
BUN (mg/dl)	31.6 ± 11.9
Cre (mg/dl)	1.6 ± 0.6
eGFR (mL/min/1.73m^2^)	32.6 ± 11.6
Na (mEql/l)	138.7 ± 3.9
K (mEql/l)	4.4 ± 0.5
Cl (mEql/l)	103.7 ± 3.8
RBC (× 104/mm3)	336.1 ± 59.1
Hb (g/dl)	10.1 ± 1.4
Hct (%)	31.2 ± 4.5
MCV (fl)	94.9 ± 7.9
Reticulo (× 104/μl)	4.7 ± 1.4
Plt (× 104/mm3)	17.7 ± 7.3
Fe (μg/dl)	67.3 ± 24.3
TSAT (%)	24.2 ± 10.0
Ferritin (ng/ml)	149.8 ± 158.5
CRP	0.9 ± 2.5
NT-proBNP (pg/ml)	1177 (642–1840)

The usage of ESA, sodium ferrous citrate, angiotensin-converting enzyme inhibitors/angiotensin-receptor blockers, beta-blockers, calcium channel blockers, statin, SGLT2 inhibitors, tolvaptan, aldosterone blockers, angiotensin receptor-Neprilysin inhibitor and diuretics at baseline was 38.5%, 38.5%, 48.7%, 71.8%, 38.5%, 71.8%, 28.2%, 60.0%, 38.5%, 23.1% and 74.8%, respectively. The rate of usage of antiplatelet drugs and anticoagulant drugs was 38.5% and 51.3% (Additional file 2: Table S1). The orally administered HIF-PH inhibitors were roxadustat, daprodustat and vadadustat, which were used in 59%, 36% and 5% respectively.

During the 3-month follow-up period of this study, events such as death, cardiovascular death, or thrombosis were not observed in any of the patients.

### Changes in hematologic status and NT-proBNP

**Fig. 1:**
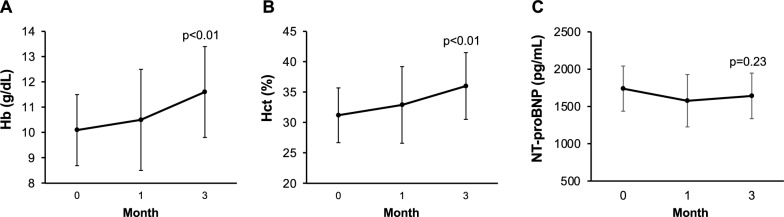
Changes in Hb (**A**), Hct (**B**), and NT-proBNP (**C**) levels during a 3-month follow-up. Data are presented as means ± SD. *Hb* hemoglobin, *Hct* hematocrit

At baseline, the mean Hb level was 10.1 ± 1.4 g/dl, and it statically increased after 3 months to 11.6 ± 1.8 g/dl (p < 0.01). At baseline, the mean hematocrit (Hct) level was 31.2 ± 4.5%. After 3 months of HIF-PHD inhibitor treatment, Hct statically increased to 36.0 ± 4.5% (p < 0.01) (Fig. 1A, B). The mean MCV and MCH levels were not statically changed during follow-up periods (94.9 ± 7.9 fl /30.3 ± 2.7 pg at baseline, and 96.7 ± 6.9 fl /31.2 ± 2.3 pg after 3 months, p = 0.24/0.06).

In addition, we assessed NT-proBNP levels, which is an indicator of heart failure. The NT-proBNP level was 1738 ± 304 pg/ml at baseline. After 3 months, NT-proBNP was not statically changed (1640 ± 306 pg/ml, p = 0.23) (Fig. 1C).

### Patient characteristics according to the changes in NT-proBNP

Next, we divided the patients into those who responded to treatment of heart failure with HIF-PH inhibitors and those who did not to evaluate various parameters. We subtracted the baseline NT-proBNP from NT-proBNP level obtained after 3 months and defined the value divided by baseline NT-proBNP as %delta NT-proBNP to evaluate the patients in this study. The median %delta NT-proBNP in our study was 4.2%. Therefore, we divided the patients into two groups using the median %delta NT-proBNP of 4.2 as the cutoff value, which allowed us to group patients with an unchanged or improved state of heart failure separately from patients with a deteriorating state of heart failure for evaluation (responder group: %delta NT-proBNP < 4.2, non-responder group: %delta NT-proBNP ≥ 4.2).

Baseline clinical characteristics of each group are shown in Additional file 2: Table S2. There were 16 patients in the responder group and 16 patients in the non-responder group. No significant differences between the two groups were observed in terms of age, gender, BMI, Hb, Hct, TSAT, ferritin, BP, EF, NT-proBNP, renal function, prevalence of disease, and concomitant medication.

### Importance of ferritin level at baseline and TSAT values after 1 month of HIF-PH inhibitor treatment

HIF-PH inhibitors are known to improve the efficiency of iron utilization [13]. TSAT of less than 20% and a ferritin level of less than 100 ng/mL were used to evaluate the absence or presence of iron utilization disorder/iron deficiency. Therefore, the numbers of patients with TSAT of less than 20% or a ferritin level of less than 100 ng/mL after the start of HIF-PH inhibitor treatment were compared between the responder and non-responder groups.

Table 2 shows the numbers of patients with TSAT of less than 20% or a ferritin level of less than 100 ng/mL at baseline and 1 and 3 months after administration of HIF-PH inhibitors. Considerably more patients had a ferritin level of less than 100 ng/mL in the non-responder group at baseline. At 1 month after the start of HIF-PH inhibitor treatment, there were considerably more patients with TSAT of less than 20% in the non-responder group. At 3 months after treatment initiation, there was no difference in the percentage of patients with either TSAT of less than 20% or a ferritin level of less than 100 ng/ml between the two groups.

**Table 2:** Correlation between changes in NT-proBNP and iron dynamics

	Responder	Non-Responder	OR (95% CI)	p-value
Baseline				
TSAT < 20	2	5	0.27 (0.04–1.73)	0.21
Ferritin < 100	4	8	0.14 (0.02–0.93)	**0.04**
1 month later				
TSAT < 20	0	5	0.09 (0.01–0.91)	**0.03**
Ferritin < 100	3	6	0.10 (0.01–1.29)	0.08
3 months later				
TSAT < 20	1	5	0.15 (0.02–1.53)	0.17
Ferritin < 100	7	9	0.56 (0.12–2.53)	0.70

p-value: Bold indicate significant

Furthermore, we performed ROC analysis to determine the cut-off point of ferritin level at baseline and TSAT at 1 month after treatment to maximize its predictive value for the non-responder group. The cut-off values and area under the curve of ferritin level at baseline were 41.8 ng/ml and 0.821 (95% confidence interval: 0.62–1.00, P < 0.01) (sensitivity 1.0 and specificity 0.6) and of TSAT at 1 month after treatment were 20.75 and 0.813 (95% confidence interval: 0.57–1.00, P = 0.04) (sensitivity 1.0 and specificity 0.625), respectively (Additional file 1: Fig. S1 and S2).

## Discussion

This study examined whether HIF-PH inhibitors were effective in patients with anemia associated with CKD who suffered from heart failure. The administration of HIF-PH inhibitors resulted in substantial increases in Hb and Hct levels after 3 months. In addition, no adverse events including thrombosis were observed during the follow-up period. Thus, the inhibitors were demonstrated to be relatively safe agents that could improve anemia.

Past large-scale clinical trials have shown that the use of ESAs to normalize Hb levels does not improve the prognosis of cardiovascular events or heart failure [5, 14]. Moreover, the PRO2TECT trial showed that HIF-PH inhibitors did not improve the prognosis of cardiovascular diseases [15]. Our results were based on short observations, and being consistent with previous results, showed that HIF-PH inhibitors did not markedly improve levels of NT-proBNP, which is an indicator of heart failure.

The HIF signal activated by HIF-PH inhibitors promotes iron absorption in the gastrointestinal tract and affects the hepcidin-ferroportin axis, thereby promoting the utilization of stored iron[16]. In other words, HIF-PH inhibitors are agents that are expected to improve iron utilization disorder. This study demonstrated that ferritin level at baseline and TSAT at 1 month after the start of HIF-PH inhibitor treatment might affect improvement of heart failure. The cut-off ferritin level at baseline and TSAT value at 1 month after the start of HIF-PH inhibitor treatment estimated by the ROC curve were 41.8 ng/ml and 20.75%, respectively. This revealed that improvement in NT-proBNP levels could be expected in patients with ferritin≧41.8 ng/ml at baseline or TSAT≧20.75 at 1 month after the start of HIF-PH inhibitor treatment. Based on the above, improving iron utilization disorder is important for improving the prognosis of heart failure, and the effect of HIF-PH inhibitors for improving TSAT may contribute to the improved prognosis of heart failure.

## Conclusion

We evaluated the safety and efficacy of HIF-PH inhibitors in non-dialysis patients with CKD and heart failure complicated by anemia. We confirmed that HIF-PH inhibitors are safe agents that could improve anemia. In addition, it was suggested that HIF-PH inhibitor treatment can be effective for improving not only anemia but also heart failure if ferritin≧41.8 ng/ml at baseline or TSAT≧20.75 at 1 month after treatment initiation.

## Limitations

This is a retrospective study conducted at a single center, and not a randomized trial; moreover, the sample size of this study is small, and an extremely large number of confounding factors can affect the changes in NT-proBNP levels. We divided the patients into two groups using the median %delta NT-proBNP of 4.2 as the cutoff value. Although a 4.2% change is relevant for the comparison groups, it may not be clinically significant. In addition, the effects of combined treatment with ESA and HIF-PH inhibitors were not examined. Finally, we examined data obtained during an extremely short follow-up period of 3 months. Therefore, long-term studies with a larger sample size have to be conducted to verify the safety and efficacy of HIF-PH inhibitors in patients with heart failure complicated by renal anemia.

### Supplementary Information

**Additional file 1.** Receiver operating characteristic curve for (Figure S1) ferritin level at baseline and (Figure S2) TSAT value at 1 month after the start of HIF-PH inhibitor treatment.

**Additional file 2: Table S1. **Comorbidities and Medication (n=32). **Table S2.** Patient characteristics according to changes in NT-proBNP.

**Additional file 3.** Additional methods.

## Data Availability

The datasets used and/or analysed during the current study are available from the corresponding author on reasonable request.

## Abbreviations

BNP: Brain natriuretic peptide
EPO: Erythropoietin
Hb: Hemoglobin
HF: Heart failure
HIF-PH: Hypoxia-inducible factor prolyl hydroxylase
Hct: Hematocrit
RDW: Red blood cell distribution width
TSAT: Transferrin saturation
