# Supplementary material for: Impact of HIF prolyl hydroxylase inhibitors in heart failure patients with renal anemia
Source: BMC Res Notes. 2024 Mar 1;17:60. doi: 10.1186/s13104-024-06726-7 (PMC10905796; doi:10.1186/s13104-024-06726-7)
Supplement: Supplementary file 1 — Additional file 1. Receiver operating characteristic curve for (Figure S1) ferritin level at baseline and (Figure S2) TSAT value at 1 month after the start of HIF-PH inhibitor treatment. [file 13104_2024_6726_MOESM1_ESM.pdf]

Figure S1

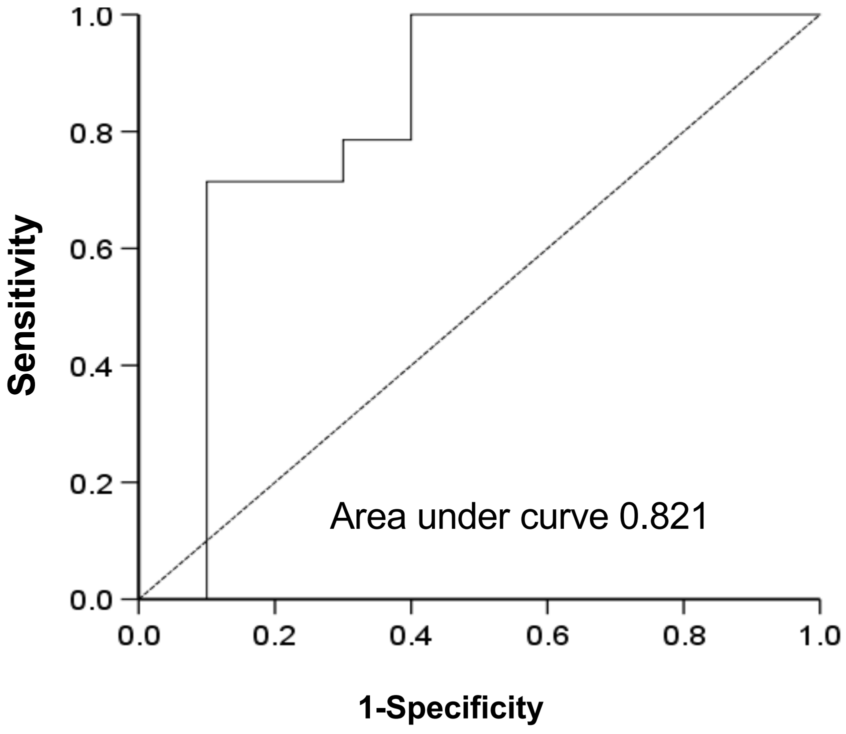

Figure S2

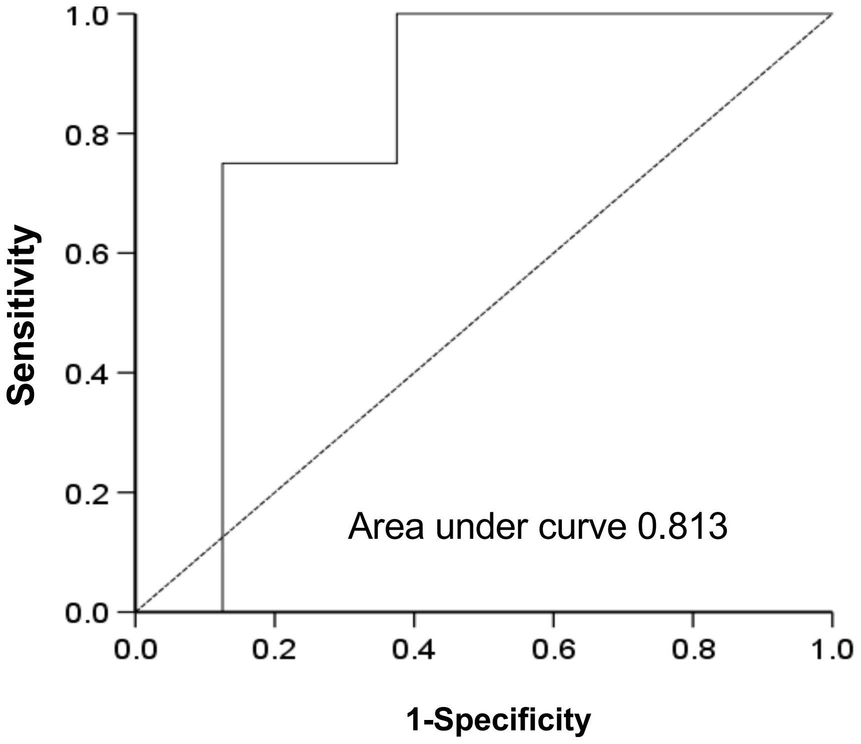

Receiver operating characteristic curve for **(Figure S1)** ferritin level at baseline and **(Figure S2)** TSAT value at 1 month after the start of HIF-PH inhibitor treatment.

Figure S1 and S2
