# Supplementary material for: Impact of HIF prolyl hydroxylase inhibitors in heart failure patients with renal anemia
Source: BMC Res Notes. 2024 Mar 1;17:60. doi: 10.1186/s13104-024-06726-7 (PMC10905796; doi:10.1186/s13104-024-06726-7)
Supplement: Supplementary file 2 — Additional file 2: Table S1. Comorbidities and Medication (n=32). Table S2. Patient characteristics according to changes in NT-proBNP. [file 13104_2024_6726_MOESM2_ESM.pdf]

Table S1. Comorbidities and Medication (n=32)

**Comorbidities** -No.(%)	
Hypertension	23 (72)
Dyslipidemia	26 (82)
Diabetes	15 (47)
Stroke	3 (9)
Coronary Artery Disease	16 (50)
Atrial Fibrillation	12 (38)
**Medication** -No.(%)	
ESA	12 (38)
Sodium ferrous citrate	11 (34)
Antiplatelet	15 (47)
Anticoagulant	17 (53)
Statin	28 (87)
ARNI	7 (22)
ACE inhibitors/ARBs	16 (50)
β-blocker	20 (63)
Calcium Channel Blocker	14 (44)
SGLT2i	11 (34)
Diuretic	29 (91)
Tolvaptan	19 (59)
Loop diuretic	22 (69)
Aldosterone Blocker	15 (47)

ESA:erythropoiesis-stimulating agent, ARNI: Angiotensin receptor-Neprilysin inhibitor, ACE, angiotensin-converting enzyme; ARBs, angiotensin receptor blockers.

Table S2. Patient characteristics according to changes in NT-proBNP

	**Responder**	**Non-Responder**	p-value
Age (years)	83±4	80±7	0.07
Male/Female	10/6	10/6	1.00
BMI (kg/cm2)	22±3	23±3	0.54
systolic BP (mmHg)	125±21	116±21	0.27
diastolic BP (mmHg)	61±13	59±10	0.64
EF (%)	59.5±10.9	53.8±13.6	0.12
HFrEF -no.(%)	1 (6)	4 (25)	0.95
HFmrEF -no.(%)	2 (13)	2 (13)	
HFpEF -no.(%)	13 (81)	10 (63)	
Hypertensions -no.(%)	12 (75)	11 (68)	0.69
Dyslipidemia -no.(%)	12 (75)	14 (88)	0.34
Diabetes -no.(%)	6 (38)	9 (56)	0.29
Stroke -no.(%)	2 (13)	1 (6)	0.60
Coronary Artery disease -no.(%)	7 (44)	9 (56)	0.70
Atrial Fibrillation -no.(%)	7 (44)	5 (31)	0.72
ESA -no.(%)	7 (44)	5 (31)	0.72
Sodium ferrous citrate -no.(%)	7 (44)	4 (25)	0.49
Hb (g/dl)	9.9±1.2	10.6±1.4	0.15
Hct (%)	31.0±3.8	33.1±4.2	0.15
TSAT (%)	27.1±6.7	21.7±9.3	0.10
Ferritin (ng/ml)	178.7±126.0	94.5±156.5	0.18
CRP (mg/dl)	1.5±3.9	0.2±0.5	0.20
BUN (mg/dl)	30.8±11.0	31.7±11.0	0.83
Cre (mg/dl)	1.5±0.5	1.7±0.8	0.36
eGFR (mL/min/1.73m^2)	35.0±12.7	31.2±10.0	0.35
Na (mEql/l)	139.1±3.8	138.5±4.5	0.70
K (mEql/l)	4.3±0.5	4.5±0.3	0.25
NT-proBNP (pg/ml)	1553 (871-3254)	867 (454-1502)	0.17
