# Supplementary material for: Impact of HIF prolyl hydroxylase inhibitors in heart failure patients with renal anemia
Source: BMC Res Notes. 2024 Mar 1;17:60. doi: 10.1186/s13104-024-06726-7 (PMC10905796; doi:10.1186/s13104-024-06726-7)
Supplement: Supplementary file 3 — Additional file 3. Additional methods. [file 13104_2024_6726_MOESM3_ESM.pdf]

Supplemental Methods

Study population

We diagnosed renal anemia in patients who had CKD with creatinine clearance of < 60, who met the following criteria for Hb levels, and who did not have white cell disorder, platelet disorder, or hemorrhagic complications: Hb levels (mg/dL) for patients aged < 60 years, < 13.5 for males and < 11.5 for females; Hb levels for patients aged 60 to < 70 years, < 12.0 for males and < 10.5 for females; and Hb levels for patients aged ≥ 70 years, < 11.0 for males and < 10.5 for females [1].

We reviewed medical records to obtain the etiology, comorbidity, and medication details. Hypertension was defined as systolic blood pressure (BP) of 140 mmHg or diastolic BP of 90 mmHg on repeated measurements or deemed present in patients undergoing antihypertensive treatment [2].

This retrospective study protocol was approved by the Ethics Committee of Tosei General Hospital, and owing to the retrospective nature of this study, the requirement of informed consent was waived based on the Japanese Ministry of Health, Labour and Welfare guidelines (Ethical Guidelines for Medical and Biological Research Involving Human Subjects: Section 8). In regard to the use of their data, patients could opt out of the study via the hospital website.

HIF-PH inhibitors

At the start of this study, three HIF-PH inhibitors, namely, roxadustat, daprodustat, and vadadustat, were available in Japan. Daprodustat interacts with CYP2C8 inhibitors and is not recommended for patients concomitantly receiving clopidogrel. Roxadustat and vadadustat have been reported to increase the blood levels of drugs that are BCRP substrates because of their BCRP-inhibitory effects. Therefore, these drugs are not recommended for patients treated with HMG-CoA reductase inhibitors. Additionally, roxadustat is administered orally three times weekly, whereas daprodustat and vadadustat are administered once daily. Based on these points, HIF-PH inhibitors were selected according to the patient’s condition and needs.

Biomarker analysis

Blood samples were obtained at each outpatient visit. Complete blood counts were performed using a Sysmex XE-5000 hematology analyzer (Sysmex, Kobe, Japan). Biochemical data were measured using a LABOSPECT 008 automatic analyzer (Hitachi Co., Tokyo, Japan). The estimated glomerular filtration rate (eGFR) was calculated using the Modification of Diet in Renal Disease formula [3].

Echocardiography

Echocardiographic examination was performed by an experienced sonographer using a Vivid E9 with XD clear imaging architecture (GE Healthcare, Tokyo, Japan). The images were recorded on the console and analyzed offline. The left ventricular ejection fraction was calculated using the modified Simpson's rule.

Follow-up and clinical outcome assessments

All patients underwent regular follow-up by outpatient clinical visits. All-cause mortality and cardiac-based mortality were determined by Tosei Hospital staff cardiologists. Cardiac-based mortality was a composite of cardiac death attributed to heart failure, myocardial infarction, or sudden death coronary heart disease. Safety was assessed on the basis of adverse events during observation periods and coded using the Medical Dictionary for Regulatory Activities (version 18.0).

Statistical analysis

All analyses were performed using PASW Statistics 18 software (SPSS Inc., Chicago, IL, USA). Continuous variables were presented as means and standard deviation (SD), and as the frequency and proportion for categorical variables. We have presented NT-proBNP levels as median and interquartile range, because these data were not normally distributed. At baseline, the differences in continuous variables between two groups were tested using the t-test. The changes in continuous variables from baseline to each time were tested using one-way repeated measures analysis of variance (one-way repeated ANOVA) with the post-hoc Dunnett's tests. Receiver operating characteristic (ROC) curve analyses were performed to identify the potential cut-off value of ferritin level at baseline and TSAT at 1 month after treatment of HIF-PH inhibitor for predicting the non-responder group. In all analyses, $p < 0.05$ was considered statistically significant.

References

1. Tsubakihara Y, Nishi S, Akiba T, Hirakata H, Iseki K, Kubota M, et al. 2008 Japanese Society for Dialysis Therapy: guidelines for renal anemia in chronic kidney disease. Ther Apher Dial 2010; 14: 240-275, doi:10.1111/j.1744-9987.2010.00836.x.

2. Umemura S, Arima H, Arima S, Asayama K, Dohi Y, Hirooka Y, et al. The Japanese Society of Hypertension Guidelines for the Management of Hypertension (JSH 2019). Hypertens Res 2019; 42: 1235-1481, doi:10.1038/s41440-019-0284-9.

3. Matsuo S, Imai E, Horio M, Yasuda Y, Tomita K, Nitta K, et al. Revised equations

for estimated GFR from serum creatinine in Japan. Am J Kidney Dis 2009; 53: 982-992, doi:10.1053/j.ajkd.2008.12.034.
